# Milk matters: seeding gut ecosystems and shaping microbiota rivalries

**DOI:** 10.20517/mrr.2024.91

**Published:** 2025-03-13

**Authors:** Laura Noël-Romas, Kelsey Fehr, Saeid Khakisahneh, Meghan B. Azad

**Affiliations:** ^1^Department of Pediatrics and Child Health, University of Manitoba, Winnipeg, MB R3E 3P4, Canada.; ^2^Manitoba Interdisciplinary Lactation Centre (MILC), Children’s Hospital Research Institute of Manitoba, Winnipeg, MB R3E 3P4, Canada.

**Keywords:** Breastfeeding, human milk, microbiome, very-low-birth-weight infants, antibiotics

## Abstract

Maternal milk contains its own diverse microbiome, which has been hypothesized to colonize the infant gut during breastfeeding; however, the dynamics of this process are not well understood, particularly among very-low-birth-weight (VLBW) infants. A recent study published in *Cell Reports Medicine* by Shama *et al.* identifies novel dose-dependent relationships between maternal milk microbiota and infant gut microbiota in a cohort of VLBW infants and further explores the potential impact of infant feeding practices and antibiotic use on these microbial colonization dynamics.

## INTRODUCTION

In their recent study, Shama *et al.* work to further our understanding of the role of breastfeeding in supporting the development of the infant gut microbiome in a high-risk population of very-low-birth-weight (VLBW) infants^[1]^. Breastfeeding and breastmilk support optimal infant development, especially in VLBW infants^[2]^. These beneficial associations are partly mediated by the gut microbiome, which is heavily influenced by maternal antibodies and the selective prebiotic effect of human milk oligosaccharides^[3]^. Additionally, breastfeeding is thought to transfer maternal milk microbes to infants in healthy term populations^[4]^. Key microbes in this process include early colonizers such as the facultative anaerobes *Staphylococcus*, *Streptococcus*, *Enterobacteriaceae*, and *Lactobacillus*, and secondary obligate anaerobes, such as *Bifidobacterium*, *Clostridium*, and *Bacteroides*, of which, *Bifidobacteria* is thought to be enriched through exclusive breastfeeding and may help control inflammatory processes to promote healthy infant development^[5]^. However, the dynamics of this microbiota sharing between maternal milk and the infant gut are not well understood, particularly among VLBW infants who may experience unique in-hospital feeding practices and increased antibiotic exposure relative to healthy term infants. A better understanding of infant gut microbiota seeding from breastmilk in VLBW infants could help guide neonatal care practices to better support infant gut microbiome colonization and, thus, overall health outcomes in these groups.

## NEW INSIGHTS ON THE TEMPORAL DYNAMICS OF MOTHER-INFANT MICROBIAL SHARING IN A VLBW POPULATION

Shama *et al.* characterized the microbiome composition of paired maternal milk and infant stool samples of 94 mother-VLBW infant dyads, which were collected across the first eight weeks postpartum from the “Optimizing Mothers Milk for Preterm Infants (OptiMoM)” trial of human milk-based fortifiers (HMBFs). Microbial species were assessed using 16S rRNA sequencing and quantitative PCR for total bacteria. Using a novel estimate of daily microbial intake from mother’s milk to determine a dose-response relationship, their findings suggest that certain microbes were able to colonize the VLBW infant gut. Notably, this colonization was stronger with direct breastfeeding and limited by antibiotic use in the postnatal period (discussed further below). The longitudinal analysis revealed that microbial diversity in mother’s milk decreases over time, while the diversity in infant stool increases, especially after initiation of direct breastfeeding. The authors further showed that the phylogenetic distance between mothers’ milk and their infants’ stool microbiota increased over time. These gradual shifts highlight an essential timeline in microbial colonization, suggesting that early interventions might capitalize on this temporal window to optimize gut health in VLBW infants.

The longitudinal nature of the data also allowed the authors to identify novel microbial community dynamics. They describe positive correlations between microbial intake from milk and infant gut microbiota at one month postpartum, which may indicate microbial colonization in an available ecological niche. However, they described an increasing number of negative correlations at two months, which may indicate increased competition in the colonization dynamics of the infant gut over time. Together, these findings reinforce and extend existing ideas regarding the importance of breastfeeding in the development of the infant gut microbiota, and this microbial exchange persists even in the context of clinical interventions that were hypothesized to disrupt both the maternal milk and infant gut flora in hospitalized VLBW infants.

## IDENTIFYING COMMONLY SHARED MICROBES USING A NOVEL METRIC OF MICROBIAL INTAKE

**Figure 1:**
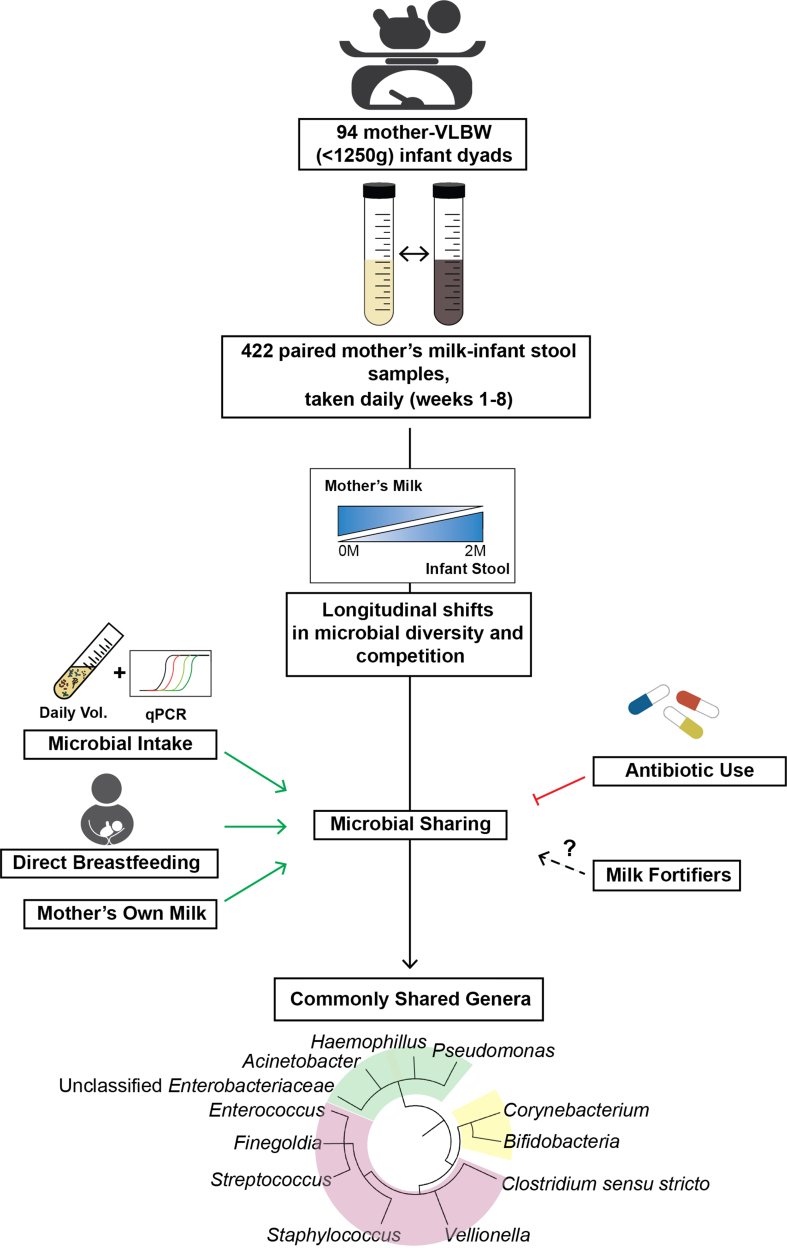
Maternal milk microbiome sharing with VLBW infants and relationships with postnatal factors. A high-level interpretation of methods and results from Shama *et al.*, 2024^[1]^. *Cell Rep Med*. 5(9):101729. VLBW: Very-low-birth-weight.

The investigators focused on commonly identified microbes within milk-stool sample pairs to estimate microbiota “sharing” and identify feeding practices to optimize this exchange. A subset of commonly shared genera between milk-stool sample pairs included the following taxa: unclassified Enterobacteriaceae, *Staphylococcus*, *Enterococcus*, *Streptococcus*, *Veillonella*, *Clostridium sensu stricto*, *Acinetobacter*, *Corynebacterium*, *Pseudomonas*, *Haemophilus*, *Finegoldia*, and *Bifidobacterium* [Figure 1]. They used a novel approach to estimate infant intake of bacteria from mother’s milk by combining quantitative PCR estimates of bacterial levels and recorded daily volume ingested by infants. This new metric will benefit future research by increasing the accuracy of microbial transfer estimates. They found a positive association between the level of these genera detected in the infant gut and intakes of these respective genera from breast milk. This positive association was strongest when the infant was fed predominantly mother’s milk (as opposed to pasteurized donor milk), indicating that ingested microbiota from breastmilk can colonize the infant gut. Additionally, the authors observed that some milk taxa were further correlated, either positively or negatively, with other taxa in the infant gut - highlighting potential cross-species interactions and community dynamics.

Interestingly, microbe sharing increased with direct breastfeeding (i.e., suckling at the mother’s breast, which is not initially possible for VLBW infants). The authors suggest that direct breastfeeding may promote the sharing of specific bacterial genera due to “backwash”, where bacteria from the infant’s oral cavity may colonize the mother’s milk and be transferred back to the infant gut. This supports previous ideas that feeding at the breast increases mother-infant microbial sharing^[7]^, adding novel information on specific genera that may be beneficial or favored in this process.

## IMPACT OF FORTIFIERS AND ANTIBIOTICS ON MOTHER-INFANT MICROBIAL SHARING

This study leveraged the unique depth of participant data available from the OptiMoM study to gain insights on how postpartum hospital practices, such as milk fortifier or antibiotic use, may influence microbe sharing over time. The findings suggest that the type of fortifier (bovine-based *vs*. human milk-based) influences the microbiota relationships between mother’s milk and infant gut, with bovine-based fortifiers unexpectedly supporting a microbiota composition that more closely mirrors the diversity of mother’s milk. This finding hints at the need for careful consideration of fortifiers, as they could modify gut microbial development in unanticipated ways, although the underlying mechanisms require further study. Further, microbial diversity was modified with antibiotic exposure. Positive relationships in shared genera between maternal milk-infant stool sample pairs were primarily observed in maternal-infant dyads with low antibiotic exposure, suggesting antibiotic use may disrupt microbial sharing. The authors emphasize the need to consider microbial sharing in antibiotic stewardship efforts for this population.

## FUTURE DIRECTIONS INSPIRED BY THIS RESEARCH

This study provides a novel characterization of mother-VLBW infant microbiome dynamics and raises several important questions for future studies:

1. Do these milk-infant microbial sharing dynamics influence infant health? Follow-up studies using these methods to define levels of infant sharing in the context of infant growth, morbidity, or mortality outcomes would be an important next step.

2. Beyond transmission and seeding - do milk microbes influence community dynamics of the infant gut microbiome? The authors focused their study on commonly identified microbes between mothers and infants; however, breastmilk microbes exist within dynamic communities, and the growth of specific taxa may promote or suppress others. Expanding the analysis to all taxa could identify broader consortium effects if, for example, unshared milk microbes could influence the sharing process without themselves being passed on to the infant.

3. How do other milk components influence milk-infant microbial sharing? Examining milk microbes in combination with other milk components, such as human milk oligosaccharides (HMOs) and maternal antibodies, along with markers of infant mucosal gut health, would provide additional context and mechanistic insights.

4. How does direct breastfeeding support microbial sharing? Several possibilities were mentioned, including the “backwash” phenomenon discussed above and the ingestion of maternal skin microbes, but further studies to confirm these mechanisms and their influence on both infant oral and gut microbial diversity and development are needed. Understanding the underlying mechanisms could help inform VLBW infant feeding and care practices to optimize microbial sharing in scenarios where direct breastfeeding is not possible.

5. Are commonly shared milk-gut microbes ideally suited to serve as oral probiotics? The commonly shared genera identified in this study, such as *Veillonella*, *Clostridium*, or *Haemophilus*, could be evaluated as new probiotic candidates tailored to support microbial maturity in preterm infants’ guts.

Taken together, this study by Shama *et al.* provides critical new information on infant gut colonization through mother’s milk in a high-risk population of infants^[1]^. These findings provide a strong basis for future studies to further investigate potential mechanisms of milk microbiota sharing and its role in both infant health and optimization of pre- and postnatal care for VLBW infants.

## DECLARATIONS

### Availability of data and materials

All relevant data and materials are available with the original publication^[1]^.

### Copyright

© The Author(s) 2025.
